# Preferential Regulation of Γ‐Secretase‐Mediated Cleavage of APP by Ganglioside GM1 Reveals a Potential Therapeutic Target for Alzheimer's Disease

**DOI:** 10.1002/advs.202303411

**Published:** 2023-09-27

**Authors:** Xiaotong Wang, Rui Zhou, Xiaqin Sun, Jun Li, Jinxin Wang, Weihua Yue, Lifang Wang, Hesheng Liu, Yigong Shi, Dai Zhang

**Affiliations:** ^1^ Peking University Sixth Hospital Peking University Institute of Mental Health NHC Key Laboratory of Mental Health (Peking University) National Clinical Research Center for Mental Disorders (Peking University Sixth Hospital) Beijing 100191 China; ^2^ Changping Laboratory Beijing 102206 China; ^3^ Beijing Frontier Research Center for Biological Structure Tsinghua‐Peking Joint Center for Life Sciences School of Life Sciences Tsinghua University Beijing 100084 China; ^4^ State Key Laboratory of Cognitive Neuroscience and Learning and IDG/McGovern Institute for Brain Research Beijing Normal University Beijing 100875 China; ^5^ PKU‐IDG/McGovern Institute for Brain Research Peking University Beijing 100871 China; ^6^ Biomedical Pioneering Innovation Center Peking University Beijing 100871 China; ^7^ Westlake Laboratory of Life Science and Biomedicine Hangzhou Zhejiang 310024 China; ^8^ Key Laboratory of Structural Biology of Zhejiang Province School of Life Sciences Westlake University Hangzhou Zhejiang 310024 China; ^9^ Institute of Biology Westlake Institute for Advanced Study 18 Shilongshan Road, Xihu District Hangzhou Zhejiang 310024 China

**Keywords:** Alzheimer's disease, conformation, ganglioside GM1, presenilin1, β‐amyloid protein

## Abstract

A hallmark of Alzheimer's disease (AD) is the senile plaque, which contains β‐amyloid peptides (Aβ). Ganglioside GM1 is the most common brain ganglioside. However, the mechanism of GM1 in modulating Aβ processing is rarely known. Aβ levels are detected by using Immunohistochemistry (IHC) and enzyme‐linked immune‐sorbent assay (ELISA). Cryo‐electron microscopy (Cryo‐EM) is used to determine the structure of γ‐secretase supplemented with GM1. The levels of the cleavage of amyloid precursor protein (APP)/Cadherin/Notch1 are detected using Western blot analysis. Y maze, object translocation, and Barnes maze are performed to evaluate cognitive functions. GM1 leads to conformational change of γ‐secretase structure and specifically accelerates γ‐secretase cleavage of APP without affecting other substrates including Notch1, potentially through its interaction with the N‐terminal fragment of presenilin 1 (PS1). Reduction of GM1 levels decreases amyloid plaque deposition and improves cognitive dysfunction. This study reveals the mechanism of GM1 in Aβ generation and provides the evidence that decreasing GM1 levels represents a potential strategy in AD treatment. These results provide insights into the detailed mechanism of the effect of GM1 on PS1, representing a step toward the characterization of its novel role in the modulation of γ‐secretase activity and the pathogenesis of AD.

## Graphical abstract

The authors show that GM1 induced conformational change of presenilin 1 (the catalytic subunit of γ‐secretase) into a preactivated state that is ready to accommodate the substrate, thus promoting γ‐secretase cleavage of APP without affecting other substrates. At last, reducing GM1 levels significantly decreases the amyloid plaque deposition and cognitive dysfunction via reducing γ‐secretase activity.

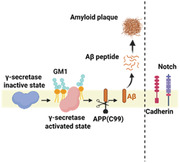

## 1 Introduction

Alzheimer's disease (AD) is a progressive neurodegenerative disorder characterized by extracellular senile plaques and age‐related impairment in cognitive functions.^[^ ^1^ ^]^ No effective clinical treatment approach has been reported to cure AD or slow its progression. Senile plaques are mainly composed of β‐amyloid (Aβ) peptides,^[^ ^2^ ^]^ which are generated through the sequential cleavage of amyloid precursor protein (APP) by β‐ and γ‐secretases.^[^ ^3^, ^4^ ^]^ Therefore, reducing Aβ production is a potential strategy for AD treatment. However, the efficacy and safety of this therapeutic approach as well as the underlying mechanisms are barely known.

γ‐Secretase cleavage is the final step in Aβ production and has attracted much attention in studies on AD. γ‐Secretase is an aspartyl protease composed of four different members including presenilin 1 (PS1), anterior pharynx‐defective 1, presenilin enhancer 2 (PEN‐2), and nicastrin.^[^ ^5^ ^]^ Among them, PS1 is the catalytic subunit of γ‐secretase. The cleavage of proteins, including APP, Notch, and N‐cadherin, by γ‐secretase, is essential for their physiological functions.^[^ ^6^, ^7^ ^]^ Drugs administered to inhibit γ‐secretase activity might also suppress the cleavage of widespread substrates concurrently, resulting in multiple side effects. Thus, instead of complete inhibition of γ‐secretase cleavage, decreasing Aβ levels without affecting other substrates represents an attractive therapeutic strategy.^[^ ^8^ ^]^ Our attention was drawn to lipid molecules because the enzymatic activity of γ‐secretase can be modulated by different kinds of lipids.^[^ ^9^ ^]^

Among these lipids, ganglioside GM1 is the most common brain ganglioside enriched on neuronal cell surfaces. GM1 has been reported to be involved in many functions associated with antineurotoxic, neurotrophic, and neuroprotective effects.^[^ ^10^, ^11^, ^12^, ^13^ ^]^ Importantly, GM1 appears to contribute to the pathophysiology of AD.^[^ ^14^ ^]^ Our previous study found that exogenous GM1 significantly promotes Aβ production in cultured cells, indicating a regulatory role for GM1 in APP proteolytic processing.^[^ ^4^ ^]^ In addition, a higher concentration of GM1 was detected in the frontal and temporal cerebral cortex of AD individuals than that in controls.^[^ ^15^, ^16^ ^]^ Increased GM1 was also observed in Aβ‐positive nerve terminals from the AD cortex and apoE4 knock‐in mouse brains with age.^[^ ^17^, ^18^ ^]^ These data indicated the involvement of GM1 in the pathophysiology of AD at early stage, possibly by facilitating amyloid plaque formation. However, the underlying molecular mechanisms remain unclear.

In addition, GM1 is a molecular marker of lipid rafts, suggesting its specific distribution on the membrane. The specific localization of GM1 may be tightly associated with the spatial segregation of γ‐secretase's substrates. The cleavage of APP has been found to occur within lipid rafts enriched with GM1, while Notch and N‐cadherin are localized out of lipid raft that lacks GM1.^[^ ^19^ ^]^ Thus, affecting GM1 levels might be a new strategy to specifically inhibit the γ‐secretase cleavage of APP.

Here we report that GM1 promotes Aβ production in mice. This effect is likely associated with the interaction of GM1 with PS1‐N terminal fragments (PS1‐NTF), which appears to induce conformational changes in γ‐secretase. Moreover, we demonstrate that either the reduction in GM1 generation by the glycosphingolipid inhibitor D‐threo‐1‐phenyl‐2‐de‐canoylamino‐3‐morpholino‐1‐propanol (D‐PDMP) or inhibition of the biological function of GM1 by cholera toxin subunit‐B (CTB) significantly reduced the Aβ levels without affecting the cleavage of other substrates by γ‐secretase. This study identifies GM1 as a potential target to specifically modulate the γ‐secretase activity towards different substrates and paves the path for developing novel strategies for AD healthcare.

## 2 Results

### 2.1 GM1 Injection Aggravates Cognitive Dysfunction of Mice

**Figure 1:**
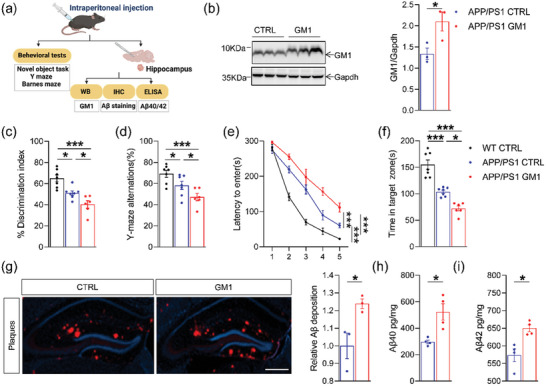
GM1 aggravated Aβ levels and cognitive dysfunction in APP/PS1 mice. a) Schematic diagram of the experimental design for the study. Behavior tests and biochemical experiments were observed after the GM1 injection. b) Western blot analysis of GM1 levels in the hippocampus (*n* = 3 per group). c) The exploratory preference to novel local objects in the object translocation test (*n* = 6–7 per group). d) Spontaneous alternations in the Y‐maze test (*n* = 6–7 per group). e,f) Escape latency during the acquisition phase (Days 1–5) and the time in target quadrant (Day 6) in the Barnes maze task (*n* = 6–7 per group). g) Aβ plaque load was labeled with an antibody directed against Aβ, and incubated with fluorescein‐conjugated secondary antibody. The images were captured by fluorescent microscopy. Image J software was used to quantify the area of hippocampus and Aβ fluorescence signals. The ratio of fluorescence signals/the area of hippocampus was assessed in each group. The data were normalized to the APP/PS1 CTRL group. Histograms showed relative changes of Aβ deposition of APP/PS1 GM1 mice compared to APP/PS1 CTRL mice. Symbols in graphs represent individual animals (*n* = 3 per group). h,i) Levels of Aβ40 and Aβ42 (pg/mg tissue) in the hippocampus were determined by ELISA)analysis (*n* = 4 per group). The data were presented as means ± SEM. Student's t‐test and two‐way ANOVA were performed for statistical analysis. *p < 0.05, **p < 0.01, ***p < 0.005. β‐amyloid peptides (Aβ), APPswe/PS1ΔE9 (APP/PS1), Enzyme‐linked immunosorbent assay (ELISA), Immunohistochemistry (IHC).

GM1 levels in the hippocampi of APPswe/PS1ΔE9 (APP/PS1) mice were higher and increased with age (Figure S1a,b, Supporting Information), indicating that GM1 plays an important role in AD pathological process.^[^ ^15^, ^16^ ^]^ To validate the involvement of GM1 in the etiology of AD, we administered GM1 to APP/PS1 mice and performed the behavioral tests and biochemical experiments (**Figure** **1**a). After injection, increased GM1 levels were detected by staining with horseradish peroxidase (HRP)‐coupled CTB in the hippocampi of 8‐month‐old APP/PS1 mice (Figure 1b, *p* = 0.0442).

We first tested the effect of GM1 on cognitive dysfunction by using object translocation test, spontaneous Y‐maze, and Barnes maze task in 8‐month‐old mice. At the training stage of the object translocation test, mice in all groups showed no significant preference for searching the two identical objects (*p* > 0.05), as evidenced by the sniff time of the objects. However, GM1‐administered APP/PS1 mice showed a decreased preference for sniffing the novel‐located object at the probe stage compared with the controls (*p* = 0.0482, Figure 1c). Although mice in all groups presented a similar pattern of arm entries in the Y‐maze (*p* > 0.05), GM1 administration significantly decreased the accurate alternation percentage compared to the controls (*p* = 0.0471, Figure 1d), indicating that GM1 treatment aggravated the impairment of working memory in APP/PS1 mice. In the Barnes maze task, GM1‐administered APP/PS1 mice spent significantly more time finding the escape box than that in APP/PS1 control (APP/PS1 CTRL) mice during training phase (Figure 1e, *p* = 0.0015). In addition, APP/PS1 mice treated with GM1 spent significantly less time in the target quadrant than APP/PS1 CTRL mice during the probe trials (Figure 1f, *p =* 0.0253).

Within expectation, GM1 administration significantly increased the total Aβ plaques in the hippocampi of APP/PS1 mice (Figure 1g, *p* = 0.0366), which was consistent with the increased Aβ40 and Aβ42 levels in hippocampal homogenates determined by enzyme‐linked immunosorbent assay (ELISA) (*p*  = 0.0100 and *p*  = 0.0117, respectively) (Figure 1h,i). These results indicate a strong correlation between increased GM1 and aggravated cognitive impairment in APP/PS1 mice, which is consistent with the elevated GM1 levels in the frontal and temporal cerebral cortex of AD patients.^[^ ^15^, ^16^ ^]^

### 2.2 Promotion of Endogenous GM1 Levels Aggravates Cognitive Dysfunction with Increasing Aβ Levels

**Figure 2:**
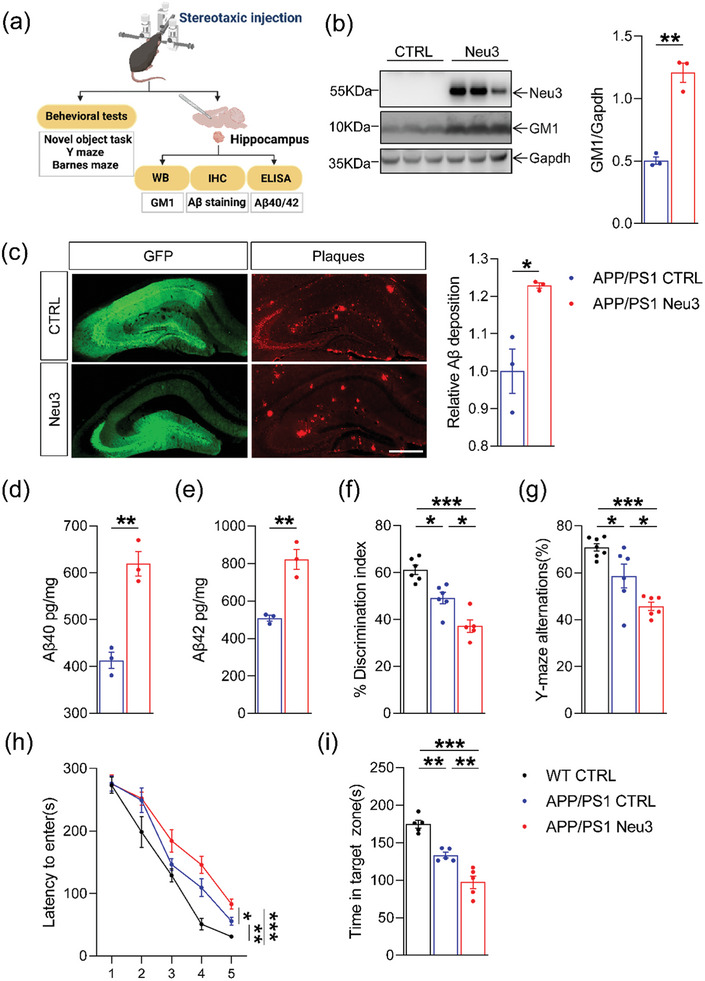
Neu3 aggravated Aβ levels and cognitive dysfunction in APP/PS1 mice. a) Schematic diagram of the experimental design for this study. AAV‐Neu3 was stereotaxically injected into the hippocampus in APP/PS1 mice at 2 months of age. Animals were tested at 8 months of age for behavior tests and biochemical experiments. b) Western blot analysis of GM1 levels in the hippocampus (*n* = 3 per group). c) Aβ plaque load was labeled with an antibody directed against Aβ, and incubated with fluorescein‐conjugated secondary antibody. The images were captured by fluorescent microscopy. Image J software was used to quantitate the area of hippocampus and Aβ fluorescence signals. The ratio of fluorescence signals/the area of hippocampus was assessed in each group. The data were normalized to the APP/PS1 CTRL group. Histograms showed relative changes of Aβ deposition of APP/PS1 GM1 mice compared to APP/PS1 CTRL mice. Symbols in graphs represent individual animals (*n* = 3 per group). d,e) The levels of Aβ40 and Aβ42 (pg/mg tissue) were determined by ELISA analysis (*n* = 3 per group). f) The exploratory preference to novel local objects in the object translocation test (*n* = 5–6 per group). g) Spontaneous alternations in the Y‐maze test (*n* = 6–7 per group). h,i) Escape latency during the acquisition phase (Days 1–5) and the time in target quadrant (Day 6) in Barnes maze task (*n* = 5 per group). The data were presented as means ± SEM and analyzed by Student's t‐test and two‐way ANOVA with Tukey's post hoc test. *p < 0.05, **p < 0.01, ***p < 0.005, “ns” means no significance (*p* > 0.05). Adeno‐associated virus expressing Neu3 (AAV‐Neu3), Green fluorescent protein (GFP), β‐amyloid peptides (Aβ), APPswe/PS1ΔE9 (APP/PS1), Enzyme‐linked immunosorbent assay (ELISA).

To further corroborate the effect of high GM1 levels on Aβ deposition and cognitive dysfunction, we tried to characterize whether increased endogenous GM1 would exacerbate the cognitive dysfunction of mice. Using an adeno‐associated virus (AAV) delivery system, we overexpressed Neu3 sialidase in the hippocampi of APP/PS1 mice (**Figure** **2**a). Neu3 sialidase would promote the generation of GM1. Under the induction by Neu3 (Figure 2b, *p* = 0.0010), the GM1 levels in the hippocampi substantially accumulated with increased deposition of Aβ plaques (Figure 2c, *p* = 0.0183). Consistently, the levels of Aβ40 and Aβ42 in hippocampal lysates were upregulated (Figure 2d,e).

The behavioral assessment showed a lower discrimination ratio of newly located objects in APP/PS1 mice than in WT control (WT CTRL) mice (Figure 2f, *p* = 0.0258). The discrimination ratio of the APP/PS1 Neu3 group was lower than that of the APP/PS1 CTRL group (Figure 2f, *p* = 0.0439). Spontaneous alternation in the Y‐maze was significantly impaired in APP/PS1 mice with Neu3 overexpression compared to control mice (Figure 2g, *p* = 0.0262). During the acquisition phase, escape latency was significantly higher in the APP/PS1 Neu3 group than in the APP/PS1 CTRL group (Figure 2h, *p* = 0.0439), indicating that APP/PS1 Neu3 mice had poorer spatial learning. Compared to APP/PS1 CTRL mice, APP/PS1 Neu3 mice showed a reduced time in the target quadrant in the probe trial, which was further reduced after Neu3 overexpression (Figure 2i, *p* = 0.0081). Thus, Neu3 overexpression aggravated cognitive impairment in APP/PS1 mice, suggesting that increased GM1 levels accelerate the progression of AD. We hypothesize that reducing the GM1 levels might alleviate the symptoms of AD.

### 2.3 D‐PDMP Administration Reduces Aβ Levels and Rescues Memory Impairment

**Figure 3:**
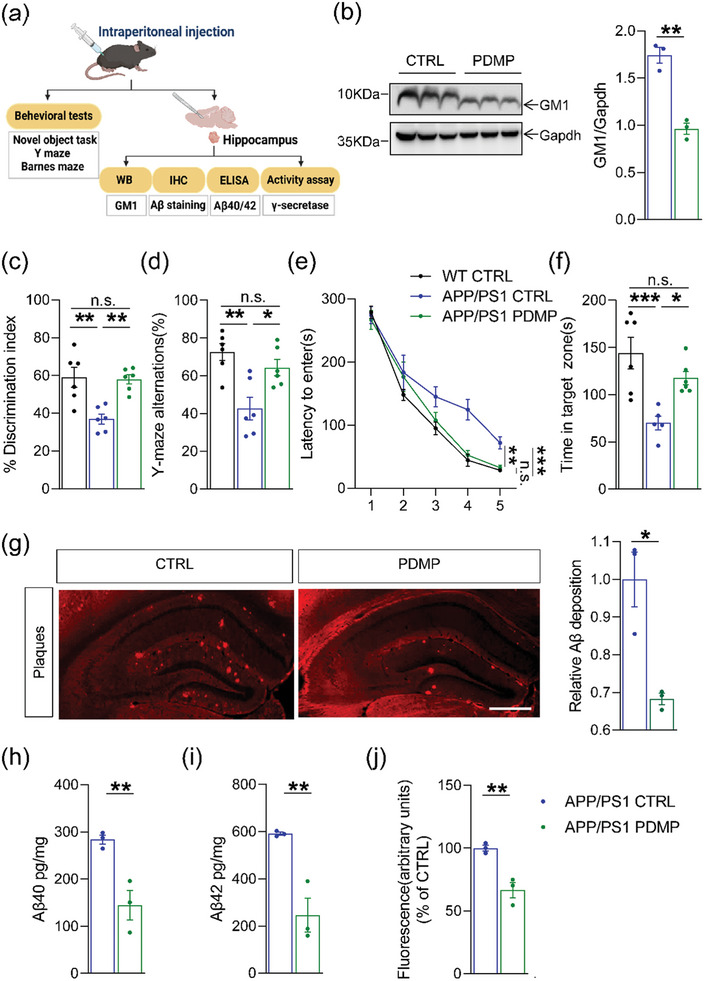
D‐PDMP treatment attenuated amyloid load and abnormal cognitive function in APP/PS1 mice. a) Schematic diagram of the experimental design for the study. Behavior tests and biochemical experiments were observed after the D‐PDMP injection. b) Western blot analysis of GM1 levels in the hippocampus (*n* = 3 per group). The levels of GM1 were normalized to the levels of Gapdh. c) The exploratory preference to novel local objects in the object translocation test (*n* = 6 per group). d) Spontaneous alternations in the Y‐maze test (*n* = 6 per group). e,f) Escape latency during the acquisition phase (Days 1–5) and the time in target quadrant (Day 6) in Barnes maze task (*n* = 5–6 per group). g) Representative images of amyloid plaque in hippocampal regions of APP/PS1 mice treated with D‐PDMP or vehicle (*n* = 3 per group). Scale bar, 500 µm. h,i) The levels of Aβ40 and Aβ42 (pg/mg tissue) in the hippocampus were determined by ELISA analysis (*n* = 3–4 per group). j) The γ‐secretase activity in membranes was measured using a fluorogenic peptide substrate as described in Methods (*n* = 3 per group). The data were presented as means ± SEM and analyzed by Student's t‐test and two‐way ANOVA with Tukey's post hoc test. *p < 0.05, **p < 0.01, ***p < 0.005, “ns” means no significance (*p* > 0.05). D‐threo‐1‐phenyl‐2‐de‐canoylamino‐3‐morpholino‐1‐propanol (D‐PDMP), β‐amyloid peptides (Aβ), APPswe/PS1ΔE9 (APP/PS1), Enzyme‐linked immunosorbent assay (ELISA).

To evaluate the effectiveness of decreased GM1 levels in preventing Aβ deposition and cognitive dysfunction, we intraperitoneally injected D‐PDMP, a glucosylceramide synthase inhibitor, into the hippocampus of APP/PS1 mice and performed the behavioral tests and biochemical experiments (**Figure** **3**a). Western blot analysis showed that the levels of GM1 significantly decreased after D‐PDMP treatment (Figure 3b, *p* = 0.0016). D‐PDMP treatment remarkably reversed the impaired discrimination ratio of the object location (Figure 3c, *p* = 0.0027). A marked increase in the percentage of accurate spontaneous alternations was observed after D‐PDMP treatment compared to the APP/PS1 CTRL group (Figure 3d, *p* = 0.0409), but was not significantly different from the WT CTRL group. Furthermore, D‐PDMP‐treated APP/PS1 mice took less time to reach the goal box than vehicle‐treated APP/PS1 mice during the training phase (Figure 3e, *p* = 0.0013) and showed significant improvement in the probe trial (Figure 3f, *p* = 0.013), indicating that the impairment of learning and memory was rescued by D‐PDMP treatment.

Accompanied with the rescued cognitive state, amyloid plaque was significantly reduced in the hippocampi of APP/PS1 mice after D‐PDMP treatment compared to the controls (Figure 3g, *p* = 0.0127). Moreover, we found a significant reduction in the levels of Aβ40 and Aβ42 after D‐PDMP treatment in APP/PS1 mice compared to the controls (Figure 3h,i; *p* = 0.0090 and *p* = 0.0046, respectively). Because Aβ peptides are generated by γ‐secretase, we analyzed the γ‐secretase activity extracted from the hippocampus. As expected, D‐PDMP greatly reduced γ‐secretase activity in the hippocampus compared to the control mice (Figure 3j, *p* = 0.0070). Together, these data suggest that reduced GM1 levels in the hippocampus is involved in the downregulation of γ‐secretase mediated Aβ processing.

### 2.4 CTB Reduces Aβ Levels and Cognitive Deficits

**Figure 4:**
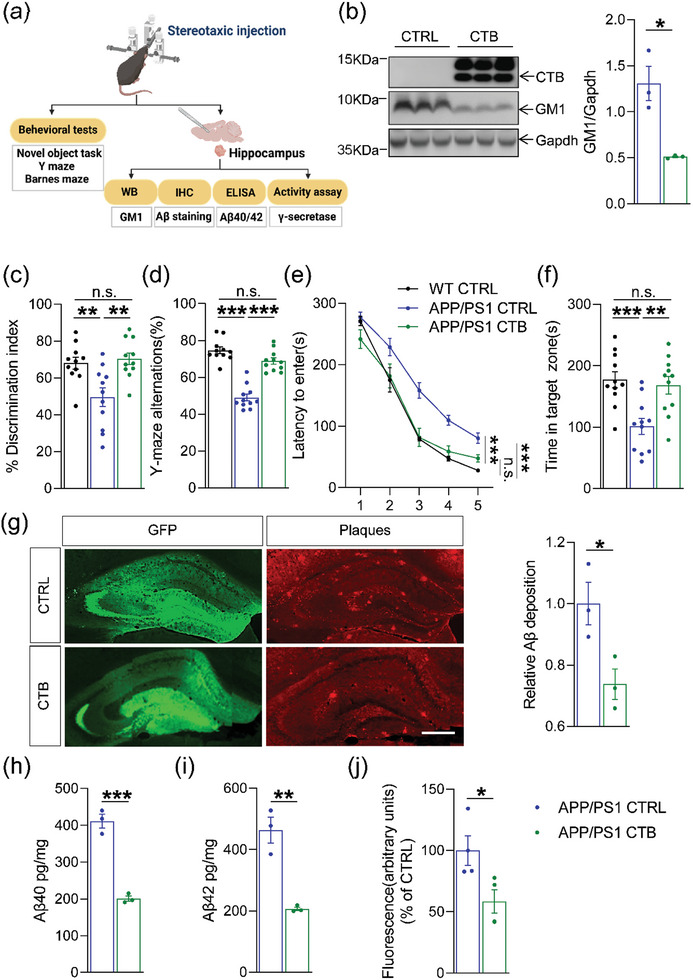
CTB attenuated amyloid load and cognitive deficits in AD‐like mice. a) Schematic diagram of the experimental design for this study. AAV‐CTB was stereotaxically injected into the hippocampus in APP/PS1 mice at 2 months of age. Animals were tested at 8 months of age for behavior tests and biochemical experiments. b) Western blot analysis of GM1 levels in the hippocampus (*n* = 3 per group). c) The exploratory preference to novel local objects in the object translocation test (*n* = 11 per group). d) Spontaneous alternations in the Y‐maze test (*n* = 11 per group). e,f) Escape latency during the acquisition phase (Days 1–5) and the time in target quadrant (Day 6) in Barnes maze task (*n* = 11 per group). g) Representative images of amyloid plaque in hippocampal regions of APP/PS1 mice treated with CTB or vehicle. Scale bar, 500 µm. The Aβ deposition were analyzed using Image J. Compared with CTRL group (n = 3 per group). h,i) The levels of Aβ40 and Aβ42 (pg/mg tissue) in the hippocampus were determined by ELISA analysis (*n* = 3 per group). j) The γ‐secretase activity in membranes was measured using a fluorogenic peptide substrate as described in Methods (*n* = 3 per group). The data were presented as means ± SEM and analyzed by Student's t‐test and two‐way ANOVA with Tukey's post hoc test. *p < 0.05, **p < 0.01, ***p < 0.005, “ns” means no significance (*p* > 0.05). Adeno‐associated virus expressing cholera toxin subunit‐B (AAV‐CTB), β‐amyloid peptides (Aβ), APPswe/PS1ΔE9 (APP/PS1), Enzyme‐linked immunosorbent assay (ELISA).

CTB recognizes and binds to GM1 with high specificity and affinity.^[^ ^20^ ^]^ To evaluate the therapeutic effect of blocking GM1 activities by CTB, we delivered and overexpressed the CTB by AAV into the hippocampus of APP/PS1 mice and performed behavioral tests and biochemical experiments (**Figure** **4**a). We found that the levels of GM1 in APP/PS1 mice were significantly decreased after CTB treatment compared to that in the controls (Figure 4b, *p* = 0.0128). In the object translocation test, CTB significantly improved the place recognition memory in APP/PS1 mice with a significantly higher place discrimination index of time spent with the object in a novel place (Figure 4c, *p* = 0.0020). After treatment with CTB, the specific alternation deficit was also significantly ameliorated (Figure 4d, *p <* 0.0001), demonstrating that CTB effectively enhanced working memory. The escape latency and memory decline in the APP/PS1 CTB group were notably reduced compared with those in the APP/PS1 CTRL group in the navigation testing and probe trial of the Barnes maze task, respectively (Figure 4e,f; *p* < 0.0001 and *p* = 0.0011, respectively). These results suggest that blocking GM1 activities by CTB mitigates memory and cognitive impairments during AD development in APP/PS1 mice.

Next, we analyzed whether CTB treatment affected the amyloid load in the hippocampi of APP/PS1 mice. 8‐month‐old APP/PS1 mice presented with obvious amyloid plaque, which was alleviated by CTB treatment (Figure 4g, *p* = 0.0367). Additionally, soluble Aβ40 and Aβ42 concentrations in the hippocampi were significantly lower in the CTB‐treated group than in the vehicle group (Figure 4h,i; *p* = 0.0004 and *p* = 0.0037, respectively). These results suggest that CTB reduces brain Aβ deposition and Aβ levels in APP/PS1 mice. Similar to the effect of D‐PDMP, CTB greatly reduced γ‐secretase activity in the hippocampus compared to the control mice (Figure 4j, *p* = 0.0353).

### 2.5 GM1 Preferentially Modulates γ‐secretase Activity Toward APP

Based on the above results, the levels of GM1 positively correlate with Aβ burden and the severity of cognitive impairment. However, the molecular basis for such correlation is not clear. Because the treatment by D‐PDMP and CTB reduced the activity of γ‐secretase, we wonder whether GM1 functions in the AD process through modulating γ‐secretase.

**Figure 5:**
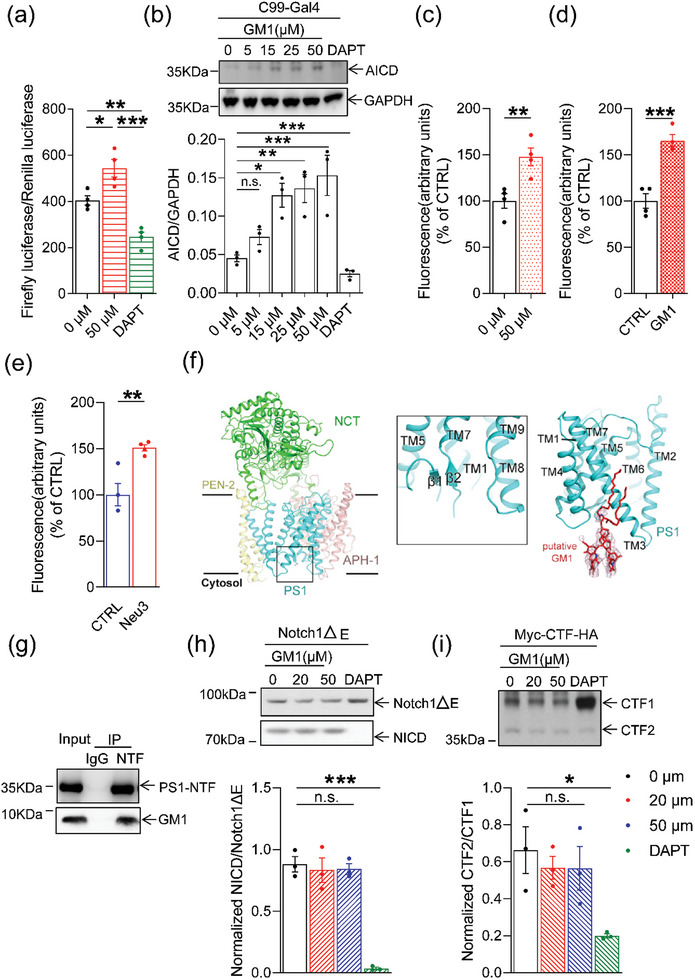
GM1 increased γ‐secretase activity through interaction with PS1. a) HeLa cells were assayed for luciferase activity with Dual‐luciferase assay system. The experiments were repeated at 4 times. b) Western blot analysis of AICD levels normalized to GAPDH. The experiments were repeated at 3 times. c–e) The γ‐secretase activity in membranes was measured using a fluorogenic peptide substrate (*n* = 3–4 per group). f) Structure of human γ‐secretase treated with GM1 determined by Cryo‐EM. The overall structure of human γ‐secretase supplemented with GM1 is shown as cartoon. Four subunits of γ‐secretase are differentially colored (left panel). Close‐up view of the intracellular β‐sheet region (middle panel). The GM1 molecule was tentatively modelled adjacent to PS1‐NTF TM3 and TM4 (right panel). Density of the putative GM1 is shown at the contour level of 5σ. g) Co‐IP analysis of endogenous PS1‐NTF and GM1. h) Immunoblots of NICD of Notch1 and quantification of the ratio of NICD/Notch1ΔE. The experiments were repeated at 3 times. i) Immunoblots of CTF1 and CTF2 of N‐Cadherin and quantification of CTF2/CTF1 of N‐Cadherin. The experiments were repeated at 3 times. The data were presented as means ± SEM and analyzed by Student's t‐test,one‐way ANOVA and two‐way ANOVA with Tukey's post hoc test. *p < 0.05, **p < 0.01, ***p < 0.005, “ns” means no significance (*p* > 0.05). Presenilin 1 (PS1), APP intracellular domain (AICD), Cryo‐electron microscopy (Cryo‐EM), PS1‐N terminal fragments (PS1‐NTF), Transmembrane (TM), Coimmunoprecipitation (Co‐IP), Notch intracellular domain (NICD), C‐terminal fragment (CTF).

We first treated N2aAPP695 cells with various concentrations of GM1. We did not observe a notable increase in the levels of full‐length APP protein after 8 h of GM1 treatment (Figure S2a, Supporting Information, *p* > 0.05). These results provide evidence that GM1 does not alter APP levels. However, Aβ40 and Aβ42 levels in the culture medium of cells treated with GM1 were greatly enhanced, as determined by ELISA (Figure S2b,c, Supporting Information; *p <* 0.0001 and *p* < 0.0001, respectively). To investigate whether GM1 improves γ‐secretase activity, we performed a luciferase reporter assay and quantitatively measured γ‐secretase cleavage of C99‐Gal4.γ‐secretase cleavage of the C99‐ Gal4 would release the APP intracellular domain (AICD), AICD‐Gal4 to promote transcription of a co‐transfected luciferase reporter gene, the activity of which can be quantified fluorometrically. GM1 treatment significantly promoted luciferase activity (**Figure** **5**a, *p* = 0.0145). The levels of the cleavage product AICD‐Gal4 were detected using Western blot analysis. The γ‐secretase inhibitor DAPT served as a control because it prevented AICD generation. As predicted, compared with the vehicle control, GM1 significantly increased AICD‐Gal4 levels (Figure 5b, *p* = 0.0003).

To assess the overall γ‐secretase activity, we isolated total cellular membranes, treated them with GM1, and conducted a fluorescence‐based activity assay.^[^ ^21^ ^]^ We found that GM1 increased the cleavage of the fluorogenic γ‐secretase peptide substrate, resulting in increased fluorescence intensity over time (Figure 5c, *p* = 0.0093), suggesting that GM1 increases γ‐secretase‐mediated cleavage of APP by directly affecting γ‐secretase activity. A fluorometric assay was performed to determine the effect of GM1 on γ‐secretase activity in vivo. The results showed that γ‐secretase activity in the hippocampus was significantly upregulated after GM1 intraperitoneal administration in APP/PS1 mice (Figure 5d, *p* = 0.0006). Consistently, γ‐secretase activity in hippocampus was significantly upregulated after Neu3 overexpression in APP/PS1 mice (Figure 5e, *p* = 0.0067).

### 2.6 GM1 Induces Conformational Changes in Human γ‐secretase

To unravel how GM1 modulates the activity of γ‐secretase, we sought to determine the 3D structure of γ‐secretase in the presence of GM1 using cryo‐electron microscopy (cryo‐EM). We finally determined the structure of WT γ‐secretase bound to GM1 at an overall resolution of 3.4 Å (Figure S3, Supporting Information). Remarkably, unlike that in the GM1‐free state,^[^ ^22^ ^]^ human γ‐secretase exhibits notable structural rearrangement in the GM1‐bound state, with a newly formed anti‐parallel β‐sheet on the intracellular side after the addition of GM1 (Figure 5f, left and middle panel; Figure S4, Supporting Information). This feature is reminiscent of the induced intermolecular β‐sheet between the substrate APP and γ‐secretase (Figure S4, Supporting Information).^[^ ^23^ ^]^ Such an observation suggests that γ‐secretase with GM1 represents a preactivated state that is ready to accommodate the substrate. This also provides a plausible explanation for why GM1 promotes γ‐secretase cleavage to a certain degree. Furthermore, upon closer inspection, a branched lamellar‐shaped density was observed near PS1‐NTF transmembrane (TM) 3 and TM4 and may correspond to the carbohydrate chain of the GM1 molecule (Figure 5f, right panel). Based on this density, a GM1 molecule was tentatively placed adjacent to the PS1 NTF (Figure 5f, right panel). However, as is often the case for bound lipid molecules, the fatty acid tail of putative GM1 lacked EM density, presumably due to flexibility.

The coimmunoprecipitation (Co‐IP) assay of the endogenous proteins in the mouse hippocampi also showed that GM1 was present in the complexes precipitated by the PS1‐NTF antibody (Figure 5g). This further verifies that GM1 interacts with PS1‐NTF and is consistent with the findings of our cryo‐EM structure.

By sharp contrast, there were no significant difference in the levels of the Notch intracellular domain (NICD) (Figure 5h) or the C‐terminal fragment CTF2/CTF1 ratio of N‐cadherin (Figure 5i, *p* > 0.05), suggesting that GM1 may specifically accelerate γ‐secretase cleavage of C99 but does not act as a general γ‐secretase activator.

## 3 Discussion

**Figure 6:**
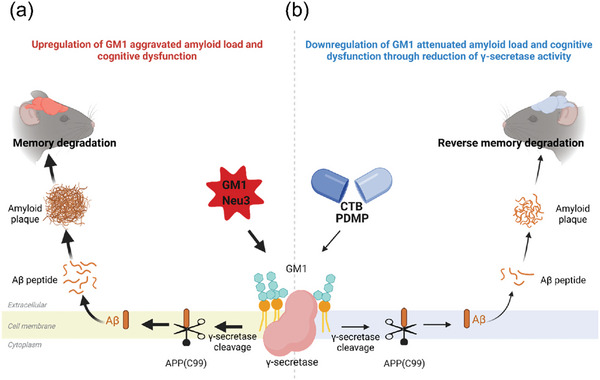
Downregulation of ganglioside GM1 decreases β‐amyloid levels and improves cognitive function by reducing γ‐secretase activity. a) Upregulation of GM1 by GM1/Neu3 increased Aβ levels and reduced cognitive function through promotion of γ‐secretase activity (left panels). b) Downregulation of GM1 by PDMP/CTB reduced Aβ levels and improved cognitive function through reduction of γ‐secretase activity (right panels).

We found that GM1 exposure leads to increased Aβ content and impaired cognitive behaviors. The increased Aβ levels might be associated with the interaction of GM1 with PS1‐NTF. Subsequently, we demonstrated that the therapeutic effect of D‐PDMP/CTB on AD might represent a novel intervention strategy to reduce Aβ levels without the side effects. The data in the current study suggest a hitherto undisclosed link between APP processing and GM1 (**Figure** **6**): GM1 promotes Aβ production and plaque deposition and aggravates cognitive impairment. Moreover, our results showed for the first time that lipid molecules, such as GM1, can induce conformational change in γ‐secretase. However, the downregulation of GM1 by D‐PDMP/CTB completely reversed these adverse effects by inhibiting γ‐secretase activity. Thus, modulating GM1 levels might be a new strategy to modulate γ‐secretase activity in AD treatment.

GM1 can readily cross the blood‐brain barrier and easily enter the brain to modulate the physiological and pathologic processes.^[^ ^24^ ^]^ Research in rodent models reported that intraperitoneal injection of GM1 increased the distribution of GM1 in lipid rafts.^[^ ^25^ ^]^ In addition, intraperitoneal injection of GM1 in mice showed that about 20%−30% of the total injected was found to stably reach the central nervous system.^[^ ^26^ ^]^ It has been demonstrated that GM1 plays a negative role in the pathophysiology of AD. The results from study on AD transgenic mouse model revealed brain region‐specific deposition of GM1 in hippocampal and cortical amyloid plaque.^[^ ^27^ ^]^ It was also shown that aging and apoE4 expression accelerates Aβ aggregation through the increase and regulation of GM1 distribution in neuronal membranes.^[^ ^17^ ^]^ These findings suggest that higher concentrations of GM1 may be associated with AD.

Furthermore, peripheral injection of GM1 impairs the cognitive performance of APP/PS1 mice.^[^ ^14^ ^]^ The most recognized mechanism is that GM1 binds to and promotes Aβ aggregation; thus, GM1 might act as a seed for Aβ deposition.^[^ ^28^, ^29^, ^30^, ^31^, ^32^, ^33^, ^34^, ^35^ ^]^ In addition to the above findings, we found a new mechanism to increase the formation of Aβ; that is, GM1 remodels the conformation of PS1 molecule, which enhances the efficiency of γ‐secretase for cleavage of APP (C99).

Previous studies have shown that decreasing the levels of GM1 with D‐PDMP^[^ ^29^, ^36^ ^]^ or blocking the GM1 sites with CTB^[^ ^37^, ^38^, ^39^ ^]^ reduced Aβ aggregation in vitro. Our study provides evidence that D‐PDMP reduces GM1 levels and Aβ production in vivo. Importantly, the coarse‐to‐fine strategy is embedded in the approach to specifically inhibit GM1 function. We demonstrated that blockade of GM1 via CTB inhibits γ‐secretase activity and Aβ production, but there is no current consensus regarding the optimal approach for CTB. In any case, blocking the biological effect of GM1 interacting with PS1 is a new strategy for the treatment of AD.

The exact mechanism by which PS1 interacts with GM1 has not yet been elucidated. Here we report the cryo‐EM structure of human γ‐secretase bound to GM1, which shows GM1‐induced conformational rearrangement of PS1 upon the addition of GM1. Identification of the putative binding site of GM1 potentially reveals the structural basis of GM1 recognition. Notably, the putative GM1 binding site is located away from the catalytic residues Asp257 on TM6 and Asp385 on TM7. However, through allostery, GM1 binding induces the formation of an anti‐parallel β‐sheet that directly stabilizes substrate binding, thus accelerating substrate cleavage. It remains to be investigated why such induced structural features work selectively for the cleavage of APP but not N‐cadherin/Notch1. One possibility is that recruitment of APP, but not N‐cadherin/Notch1, requires assistance due to its lower binding affinity. Regardless of this speculation, our structural data provide insights into the regulatory basis of GM1 on Aβ generation. In summary, although future work is necessary to determine the detailed mechanism of the effect of GM1 on PS1, our data represent a step toward the characterization of its novel role in the modulation of γ‐secretase activity and the pathogenesis of AD.

## 4 Experimental Section

### Ethics Statement

All animal procedures were approved by the Animal Ethical Committee of Peking University Health Center (LA2015114) and performed in accordance with all relevant ethical regulations.

### Animals

Male APP/PS1 double‐transgenic mice (Jackson Laboratory, stock NO.004462) (*n* = 49) and littermate wild‐type (WT) male mice (*n* = 49) aged 8‐month‐old with body weights of 30±5 g were used in this study. All mice were purchased from the Model Animal Research Center of Nanjing University (Nanjing, China). Mutations in APP and PS1 were confirmed in APP/PS1 mice using polymerase chain reaction, as previously described. Animals were maintained on a 12 h light/dark cycle with access to food and water ad libitum. After the behavioral tests, mice were sacrificed by cervical displacement.

### Intraperitoneal Drug Administration

Mice were intraperitoneally injected with GM1 (30 mg kg^−1^) diluted in saline (0.9%) daily for 28 days.^[^ ^40^, ^41^, ^42^ ^]^ GM1 was purchased from Qilu Pharmaceutical Co., (Shandong, China). Animals were selected randomly to form different groups: WT CTRL, APP/PS1 CTRL, and APP/PS1 GM1. According to the mouse body weight, one intraperitoneal injection of 30 mg kg^−1^ D‐PDMP (P7340, Sigma‐Aldrich, Tokyo, Japan), D‐PDMP powder was dissolved in saline containing 5% Tween 80.^[^ ^43^ ^]^ And the mice were divided into three subgroups: WT CTRL, APP/PS1 CTRL, and APP/PS1 D‐PDMP.

### Stereotactic Viral Microinjection

Stereotaxic surgery and AAV injection were performed as previously described.^[^ ^44^ ^]^ Mice were anesthetized by intraperitoneal injection of pentobarbital sodium (50 mg kg^−1^)^[^ ^45^, ^46^ ^]^ and placed on a stereotaxic apparatus (RWD Science). The construct was packaged into a chimeric AAV2/8 vector and was generated by OBiO Technology. A pAAV‐CMV‐NEU3‐MYC‐SFLAG‐P2A‐EGFP vector (AAV‐NEU3), A pAAV‐CMV‐CTB‐MYC‐BGHpolyA‐EF1A‐EGFP‐3FLAG vector, and a control pAAV‐CMV‐MCS‐EGFP‐3FLAG vehicle (AAV‐CTRL) (titers > 1.0 × 10^12^) were delivered via bilateral stereotactic injections into the hippocampus of the brain (0.5 µL/coordinate) following the mouse brain atlas (anterior/posterior [AP]: −1.70 mm, −2.54 mm; medial/lateral [ML]: ±1.4 mm, ±2.45 mm, dorsal/ventral [DV]: −1.8 mm, −2.1 mm). The infusions were performed at a rate of 0.1 µL min^−1^. An additional minute was allowed for diffusion and prevention of backflow through the needle track before the injector was withdrawn. The location of the virus transfection was confirmed by fluorescence.

### Cell Culture and Treatment

HEK293T and Hela cells were purchased from American type culture collection (Manassas, VA, USA). The N2a APP695 cells were purchased from Cell Resource Center, Peking Union Medical College. HEK293T and Hela cells were cultured under standard conditions (37°C, 5% CO2, 95% air) in Advanced DMEM supplemented with 10% (vol/vol) FBS, penicillin (100 U mL^−1^), streptomycin (100 µg mL^−1^) and Primocin (100 µg mL^−1^). N2a cells overexpressing human APP695 cells (N2aAPP695) were cultured in the same media as the HEK293T cells supplemented with 250 µg mL^−1^ of G418 as a selection agent. N2aAPP695 cells on a 100 mm dish grown to 85–90% confluence were washed with phosphate‐buffered saline (PBS) before treatment with 0, 10, 20, 30, 40 and 50 µm GM1 for 8 h. Then the supernatants from each group of the N2aAPP695 cells were collected.

### Dual‐Luciferase Reporter Assay

For the dual‐luciferase reporter assay, HeLa cells were cultured in 24 well plastic tissue culture plates for 1 day before transfection. A gene carrying C99‐Gal4 Eukaryotic expression recombinant plasmid (The 99 amino acids at the carboxyl terminal of human APP was cloned in the pcDNA3.1 plasmid) was constructed. HeLa cells were co‐transfected with 0.32 µg C99‐ Gal4 plasmid, 0.32 µg pGL4.35 plasmid (E1370;Promega , Madison, USA) and 0.008 µg pRL‐TK plasmid (E2241;Promega, Madison, USA) with Lipofectamine 2000 reagent (Invitrogen, CA, USA) according to manufacturer's instructions, and 6 h later, GM1 with the concentration of 50 µm, and DAPT with the concentration of 1 µm were added into the HeLa cells, following harvesting of these cells for the luciferase activity assay. A dual‐luciferase reporter assay kit (E1910; Promega, Madison, USA) was used to assess the relative luciferase activity in the dual‐luciferase reporter assay system, and Renilla luciferase activity was used for normalization of firefly luciferase activity. Three replicate lysates were analyzed in each experiment.

### γ‐secretase Activity Assay

A γ‐secretase activity assay that uses a fluorogenic peptide substrate corresponding to the APP γ‐secretase cleavage site was used. Plasma membrane samples were extracted using the plasma membrane protein isolation and cell fractionation kit (SM‐005‐50; Invent biotechnologies, Plymouth, MN, USA), according to the manufacturer's protocol. Briefly, total plasma membrane samples were resuspended on a rotator in lysis buffer (20 mm Hepes, 2 mm EGTA,150 mm KCL, 1% CHAPSO, and protease inhibitors) for 3 h at 4 °C. Solubilized samples were cleared at 12 000 g for 1 h at 4 °C. Then, supernatant (total membrane) was collected. The protein concentration was determined using Bicinchoninic acid, and 200 µg of protein was used for the γ‐secretase activity assay. The γ‐secretase activity was assayed in 50 m Tris‐HCl (pH 6.8), 4 mm EDTA, and 0.5% CHAPSO(w/v) with 3 µg fluorogenic substrate (565764; Calbiochem, San Diego, CA, USA). After incubation, the fluorescence intensities of γ‐secretase were measured at excitation wavelengths of 355 and 440 nm.

### ELISA

To measure Aβ40 and Aβ42 levels in hippocampus of APP/PS1 mouse, the tissue was homogenized in lysis buffer containing protease inhibitor cocktail and centrifuged at 12 000 g for 20 min at 4 °C. Afterward, the supernatant was isolated. The concentrations of Aβ40 and Aβ42 were measured using enzyme‐linked immunosorbent assay (ELISA) kits (Invitrogen, CA, USA. KHB3481, Aβ40; Invitrogen, CA, USA, KHB3441, Aβ42) according to the manufacturer's instructions. Absorbance was determined at 450 nm using a microplate absorbance reader.

### Immunoprecipitation

In brief, for the immunoprecipitation, lysates were obtained by sonicating the hippocampus or cells in extraction buffer containing 20 mm Tris‐HCl (pH 8), 137 mm NaCl, 0.1% Triton, and 1 mm EDTA, supplemented with a protease inhibitor. The antibodies or control immunoglobulin G and Dynabeads Protein G (10004D; Invitrogen, Carlsbad, CA, USA), respectively, were sequentially added to the precipitation system and rotated at 4 °C overnight. Protein complexes were eluted several times with a washing buffer (phosphate buffered saline, pH 7.4, containing 0.02% Tween‐20), and the immunoprecipitated proteins were ready to use for Western blot analysis.

### Object Translocation Test

The object translocation test based on the spontaneous tendency of rodents to explore novelty was performed as previously described.^[^ ^47^ ^]^ Briefly, the animals were tested in two trials. In the 10‐minute sample phase, mice interacted with two copies of an identical object, after which animals were placed back into their cage. One hour later, the mice underwent a 10‐minute test phase in which they were placed in the chamber with the two same objects, but with one relocated to a novel location, the novel location was at the opposite corner of the previous location. Each animal was videotaped by overhead cameras and scored for the total time spent investigating the objects per trial.

### Y‐Maze Task

Spatial working memory of mice was assessed by using Y‐maze test.^[^ ^48^ ^]^ The Y‐maze test involved animals freely exploring the three arms of the Y‐maze for 10 min. Appropriate cues were then assigned to each arm. All animal activities were recorded using a video camera, and the numbers of arm entries and triads were determined to calculate the percentage of alternation.

### Barnes Maze

The Barnes maze test was performed according to previous study.^[^ ^49^ ^]^ The training procedure included one trial per day for 5 consecutive days. At the start of each training trial, the mice were placed inside a start tube at the center of the platform for 15 s and then released. After entering the escape box, the mice remained there for 30 s before returning to their home cage. The escape latency was recorded and scored as a measure of acquisition. A probe test consisting of a 300‐second free exploration period without the escape box was conducted on day 6. The exploration time spent in the target quadrant was determined.

### Sample Preparation for Cryo‐EM Study

The plasmid containing four γ‐secretase components (PEN‐2 has a N‐terminal Flag tag) was transfected into HEK293F cells. After 48 h, the transfected cells were harvested by centrifugation and resuspended by lysis buffer (25 mm HEPES pH 7.4, 150 mm NaCl). The harvested cells were then applied to sonication to disrupt the cell membrane. The suspension was centrifuged at 150 000 g for 1 h to collect the membrane fraction. After incubation with 1% CHAPSO for 2 h, the cell membrane was centrifuged at 150 000 g for 30 min and the supernatant was then applied to anti‐Flag M2 gel. The resin was washed with buffer A (25 mm HEPES pH 7.4, 150 mm NaCl and 0.1% digitonin) and eluted with buffer B (25 mm HEPES pH 7.4, 150 mm NaCl, 0.1% digitonin and 200 µg mL^−1^ Flag peptide). The eluted protein was concentrated and purified by gel filtration chromatography. The peak fractions were collected and further concentrated to ≈40 µM for cryo‐EM study. The concentrated protein was incubated with a final concentration of 0.5 mg mL^−1^ (0.34 mm) GM1 molecule for 1 h at 4 °C. The cryo‐EM grids for data collection were made using a Vitrobot (FEI). Aliquots of 4 µL of GM1‐protein mixture were added to glow‐discharged grids (Quantifoil Au R1.2/1.3). The grids were blotted for 3 s and frozen by liquid ethane, and then transferred to liquid nitrogen for storage.

### Data Collection and Processing for Cryo‐EM Analysis

The prepared grids were imaged on an FEI 300 kV Titan Krios electron microscope equipped with GIF Quantum energy filter (slit width 20 eV) and Gatan K3 Summit detector with a nominal magnification of 64000 × (pixel size 1.0979). In total, 4031 micrographs were collected. MotionCor2 and Gctf were used for motion correction and defocus value estimation, respectively. 1, 097, 691 particles were auto‐picked with RELION (version 3.0) and were subjected to 2D classification. 409, 964 particles were selected for 25 iterations of global angular search 3D classification with a class number of 1 and step size of 7.5°. For the last six iterations (No. 20–25) of the global search, the local angular search 3D classification was performed with a class number of four, a step size of 3.75°, and a local search range of 15°. For the last iteration of the local search, particles from the good classes were merged and duplicated particles were removed. The merged 297134 particles were auto‐refined, yielding 3D reconstructions at 4.2 Å. Then, multi‐reference models were generated from one of the last iterations of local search 3D classification. The particles from the initial refinement were applied to multi‐reference‐based 3D classification. Finally, 134908 particles from the good classes were auto‐refined and postprocessed, resulting in 3.4‐Å reconstruction based on the Fourier shell correlation (FSC) 0.143 criterion. Local resolution estimation was performed using RELION‐3.0.

### Antibodies

The following antibodies were used: amyloid (D54D2) (8243s,1:500; Cell Signaling Technology, MA, USA), Myc (3956, 1:1000; Sigma‐Aldrich, Tokyo, Japan), anti‐amyloid precursor protein, C‐terminal antibody (A8717, 1:1000; Sigma‐Aldrich, Tokyo, Japan), PS1 (5643s, 1:1000; Cell Signaling Technology, MA, USA), cadherin (3195s, 1:1000; Cell Signaling Technology, MA, USA), cleaved‐Notch 1 (4147s, 1:1000; Cell Signaling Technology, MA, USA), anti‐GAPDH (5174, 1:3000; Cell Signaling Technology, MA, USA), Alexa 555 goat anti‐mouse (A21422, 1:500; Invitrogen, OR, USA), and Alexa 488 goat anti‐rabbit (A11008, 1:500; Invitrogen, OR, USA).

### Amyloid Counts

Quantification of β‐amyloid plaque was carried out on IHC images captured from sections stained with anti‐Aβ antibody. IHC images were first transformed to monochrome and inverted to make amyloid plaque bright areas. Sections of each mouse brain were imaged, and the areas and densities of the plaques present in the hippocampus brain regions were measured. An intensity threshold level was established to discriminate between plaque immunostaning and background labeling. The threshold for detection was held constant throughout the image quantification. An object of interest was defined by its surface and its brightness.

### Statistical Analysis

The normal distribution of the data was tested to verify the use of parametric or non‐parametric statistical analysis. Data were first examined for assumption of normality using the Shapiro‐Wilk statistic and for homogeneity of variance using the Levene's test. All data are presented as mean ± standard error of the mean. The Student t‐test (two‐tailed) was performed to detect significant differences between the two groups. Differences between multiple groups were analyzed using one‐way and two‐way analysis of variance for one independent variable and two independent variables, respectively, followed by the post hoc Tukey test. The sample size (n) values are provided in the relevant text, figures, and figure legends. All statistical analyses were performed using GraphPad Prism software (version 8.0; GraphPad Software). Statistical significance was set at *p < 0.05, **p < 0.01, ***p < 0.005.

## Conflict of Interest

The authors declare no conflict of interest.

## Author Contributions

X.W., R.Z., and X.S. contributed equally to this work. D.Z., Y.S., and R.Z. contributed to the funding acquisition, provided the resources, designed this research, and edited the manuscript. X.W. performed experiments, analyzed data, and wrote the manuscript. J.L. designed the experiments and wrote the manuscript. X.S. performed the experiments and analyzed data. R.Z. and Y.S. designed and performed the cryo‐electron microscopy experiments and analyzed data. J.W., W.Y., L.W., and H.L. provided technical support.

## Supporting information

Supporting Information

## Data Availability

The data that support the findings of this study are available from the corresponding author upon reasonable request.

## References

[1] V. L. Villemagne , S. Burnham , P. Bourgeat , B. Brown , K. A. Ellis , O. Salvado , C. Szoeke , S. L. Macaulay , R. Martins , P. Maruff , D. Ames , C. C. Rowe , Lancet Neurol. 2013, 12, 357. 23477989 10.1016/S1474-4422(13)70044-9

[2] J. Hardy , D. J. Selkoe , Science 2002, 297, 353. 12130773 10.1126/science.1072994

[3] G. Barthet , A. Georgakopoulos , N. K. Robakis , Prog Neurobiol 2012, 98, 166. 22622135 10.1016/j.pneurobio.2012.05.006 PMC3404154

[4] H. Zhang , Q. Ma , Y. W. Zhang , H. Xu , J. Neurochem. 2012, 120, 9. 22122372 10.1111/j.1471-4159.2011.07519.x PMC3254787

[5] G. Thinakaran , D. R. Borchelt , M. K. Lee , H. H. Slunt , L. Spitzer , G. Kim , T. Ratovitsky , F. Davenport , C. Nordstedt , M. Seeger , J. Hardy , A. I. Levey , S. E. Gandy , N. A. Jenkins , N. G. Copeland , D. L. Price , S. S. Sisodia , Neuron 1996, 17, 181. 8755489 10.1016/s0896-6273(00)80291-3

[6] A. Haapasalo , D. M. Kovacs , J Alzheimers Dis 2011, 25, 3.21335653

[7] J. L. Ables , J. J. Breunig , A. J. Eisch , P. Rakic , Nat Rev Neurosci 2011, 12, 269. 21505516 10.1038/nrn3024 PMC3159580

[8] D. J. Selkoe , Nat. Med. 2011, 17, 1060. 21900936 10.1038/nm.2460

[9] E. Dawkins , R. J. E. Derks , M. Schifferer , J. Trambauer , E. Winkler , M. Simons , D. Paquet , M. Giera , F. Kamp , H. Steiner , J Biol Chem 2023, 299, 103027. 36805335 10.1016/j.jbc.2023.103027 PMC10070668

[10] M. Aureli , L. Mauri , M. G. Ciampa , A. Prinetti , G. Toffano , C. Secchieri , S. Sonnino , Mol. Neurobiol. 2016, 53, 1824. 25762012 10.1007/s12035-015-9136-z

[11] R. W. Ledeen , G. Wu , Trends Biochem. Sci. 2015, 40, 407. 26024958 10.1016/j.tibs.2015.04.005

[12] P. J. Magistretti , F. H. Geisler , J. S. Schneider , P. A. Li , H. Fiumelli , S. Sipione , Front Neurol 2019, 10, 859. 31447771 10.3389/fneur.2019.00859 PMC6691137

[13] C. L. Schengrund , Trends Biochem. Sci. 2015, 40, 397. 25941169 10.1016/j.tibs.2015.03.007

[14] C. C. Yang , Y. Cheng , H. M. Yang , Y. Chen , Y. J. Wang , Z. Q. Xu , Y. R. Wang , Neurosci. Bull. 2022, 38, 95. 34510368 10.1007/s12264-021-00768-8 PMC8782970

[15] M. Molander‐Melin , K. Blennow , N. Bogdanovic , B. Dellheden , J. E. Mansson , P. Fredman , J. Neurochem. 2005, 92, 171. 15606906 10.1111/j.1471-4159.2004.02849.x

[16] Z. Pernber , K. Blennow , N. Bogdanovic , J. E. Mansson , M. Blomqvist , Dement. Geriatr. Cogn. Disord. 2012, 33, 174. 22572791 10.1159/000338181

[17] N. Yamamoto , U. Igbabvoa , Y. Shimada , Y. Ohno‐Iwashita , M. Kobayashi , W. G. Wood , S. C. Fujita , K. Yanagisawa , FEBS Lett. 2004, 569, 135. 15225622 10.1016/j.febslet.2004.05.037

[18] K. H. Gylys , J. A. Fein , F. Yang , C. A. Miller , G. M. Cole , Neurobiol. Aging 2007, 28, 8. 16332401 10.1016/j.neurobiolaging.2005.10.018

[19] K. S. Vetrivel , H. Cheng , S. H. Kim , Y. Chen , N. Y. Barnes , A. T. Parent , S. S. Sisodia , G. Thinakaran , J. Biol. Chem. 2005, 280, 25892. 15886206 10.1074/jbc.M503570200 PMC1201532

[20] I. Kracun , S. Kalanj , J. Talan‐Hranilovic , C. Cosovic , Neurochem. Int. 1992, 20, 433. 1304338 10.1016/0197-0186(92)90058-y

[21] T. Sarajarvi , J. T. Tuusa , A. Haapasalo , J. J. Lackman , R. Sormunen , S. Helisalmi , J. T. Roehr , A. R. Parrado , P. Makinen , L. Bertram , H. Soininen , R. E. Tanzi , U. E. Petaja‐Repo , M. Hiltunen , Mol. Cell. Biol. 2011, 31, 2326. 21464208 10.1128/MCB.05015-11 PMC3133236

[22] X. C. Bai , C. Yan , G. Yang , P. Lu , D. Ma , L. Sun , R. Zhou , S. H. W. Scheres , Y. Shi , Nature 2015, 525, 212. 26280335 10.1038/nature14892 PMC4568306

[23] R. Zhou , G. Yang , X. Guo , Q. Zhou , J. Lei , Y. Shi , Science 2019, 363, 708. 10.1126/science.aaw0930 30630874

[24] E. Di Biase , G. Lunghi , M. Maggioni , M. Fazzari , D. Y. Pome , N. Loberto , M. G. Ciampa , P. Fato , L. Mauri , E. Sevin , F. Gosselet , S. Sonnino , E. Chiricozzi , Int. J. Mol. Sci. 2020, 21, 2858. 32325905 10.3390/ijms21082858 PMC7215935

[25] Y. P. Zhang , Q. L. Huang , C. M. Zhao , J. L. Tang , Y. L. Wang , Braz. J. Med. Biol. Res. 2011, 44, 553. 21670940 10.1590/s0100-879x2011000600009

[26] E. Chiricozzi , L. Mauri , G. Lunghi , E. Di Biase , M. Fazzari , M. Maggioni , M. Valsecchi , S. Prioni , N. Loberto , D. Y. Pome , M. G. Ciampa , P. Fato , G. Verlengia , S. Cattaneo , R. Assini , G. Wu , S. Alselehdar , R. W. Ledeen , S. Sonnino , Sci. Rep. 2019, 9, 19330. 31852959 10.1038/s41598-019-55885-2 PMC6920361

[27] I. Kaya , E. Jennische , J. Dunevall , S. Lange , A. G. Ewing , P. Malmberg , A. T. Baykal , J. S. Fletcher , ACS Chem. Neurosci. 2020, 11, 14. 31774647 10.1021/acschemneuro.9b00532

[28] K. Yanagisawa , A. Odaka , N. Suzuki , Y. Ihara , Nat. Med. 1995, 1, 1062. 7489364 10.1038/nm1095-1062

[29] N. Yamamoto , Y. Fukata , M. Fukata , K. Yanagisawa , Biochim. Biophys. Acta 2007, 1768, 1128. 17306220 10.1016/j.bbamem.2007.01.009

[30] K. Matsuzaki , K. Kato , K. Yanagisawa , Biochim. Biophys. Acta 2010, 1801, 868. 20117237 10.1016/j.bbalip.2010.01.008

[31] M. Wakabayashi , T. Okada , Y. Kozutsumi , K. Matsuzaki , Biochem. Biophys. Res. Commun. 2005, 328, 1019. 15707979 10.1016/j.bbrc.2005.01.060

[32] L. P. Choo‐Smith , W. K. Surewicz , FEBS Lett. 1997, 402, 95. 9037173 10.1016/s0014-5793(96)01504-9

[33] H. Hayashi , N. Kimura , H. Yamaguchi , K. Hasegawa , T. Yokoseki , M. Shibata , N. Yamamoto , M. Michikawa , Y. Yoshikawa , K. Terao , K. Matsuzaki , C. A. Lemere , D. J. Selkoe , H. Naiki , K. Yanagisawa , J. Neurosci. 2004, 24, 4894. 15152051 10.1523/JNEUROSCI.0861-04.2004 PMC6729458

[34] N. Yamamoto , T. Matsubara , T. Sato , K. Yanagisawa , Biochim. Biophys. Acta 2008, 1778, 2717. 18727916 10.1016/j.bbamem.2008.07.028

[35] T. Okada , M. Wakabayashi , K. Ikeda , K. Matsuzaki , J. Mol. Biol. 2007, 371, 481. 17582434 10.1016/j.jmb.2007.05.069

[36] J. Inokuchi , N. S. Radin , J. Lipid Res. 1987, 28, 565. 2955067

[37] P. W. Janes , S. C. Ley , A. I. Magee , J. Cell Biol. 1999, 147, 447. 10525547 10.1083/jcb.147.2.447 PMC2174214

[38] H. Lin , E. N. Kitova , J. S. Klassen , J. Am. Soc. Mass Spectrom. 2014, 25, 104. 24122305 10.1007/s13361-013-0751-5

[39] A. C. Robu , Z. Vukelic , C. Schiopu , F. Capitan , A. D. Zamfir , Anal. Biochem. 2016, 509, 1. 27311552 10.1016/j.ab.2016.06.005

[40] M. Hadjiconstantinou , N. H. Neff , Ann. N. Y. Acad. Sci. 1998, 845, 225. 9668356 10.1111/j.1749-6632.1998.tb09675.x

[41] J. S. Schneider , A. Yuwiler , Exp Neurol 1989, 105, 177. 2568945 10.1016/0014-4886(89)90117-9

[42] J. S. Schneider , R. Aras , C. K. Williams , J. B. Koprich , J. M. Brotchie , V. Singh , Sci. Rep. 2019, 9, 8362. 31182727 10.1038/s41598-019-42847-x PMC6557812

[43] H. Fujiwara , K. Ikarashi , Y. Yamazaki , J. Goto , K. Kaneko , M. Sugita , H. Kato , H. Sasaki , J. Inokuchi , K. Furukawa , S. Fujii , Biomed. Res. 2012, 33, 265. 23124246 10.2220/biomedres.33.265

[44] R. J. Ernst , T. P. Krogager , E. S. Maywood , R. Zanchi , V. Beranek , T. S. Elliott , N. P. Barry , M. H. Hastings , J. W. Chin , Nat. Chem. Biol. 2016, 12, 776. 27571478 10.1038/nchembio.2160 PMC5215917

[45] J. W. Dutton 3rd , J. E. Artwohl , X. Huang , J. D. Fortman , J. Am. Assoc. Lab. Anim. Sci. 2019, 58, 373. 30857577 10.30802/AALAS-JAALAS-18-000094 PMC6526499

[46] M. Suzuki , T. Funabiki , S. Hori , N. Aikawa , Resuscitation 2009, 80, 109. 18951685 10.1016/j.resuscitation.2008.08.013

[47] M. S. Costa , P. H. Botton , S. Mioranzza , D. O. Souza , L. O. Porciuncula , Neuroscience 2008, 153, 1071. 18436387 10.1016/j.neuroscience.2008.03.038

[48] M. Ohno , E. A. Sametsky , L. H. Younkin , H. Oakley , S. G. Younkin , M. Citron , R. Vassar , J. F. Disterhoft , Neuron 2004, 41, 27. 14715132 10.1016/s0896-6273(03)00810-9

[49] I. Lena , M. Mantegazza , Sci. Rep. 2019, 9, 12886. 31501495 10.1038/s41598-019-49392-7 PMC6733925

