# Supplementary material for: Preferential Regulation of Γ‐Secretase‐Mediated Cleavage of APP by Ganglioside GM1 Reveals a Potential Therapeutic Target for Alzheimer's Disease
Source: Adv Sci (Weinh). 2023 Sep 27;10(32):2303411. doi: 10.1002/advs.202303411 (PMC10646247; doi:10.1002/advs.202303411)
Supplement: Supplementary file 1 — Supporting Information [file ADVS-10-2303411-s001.pdf]

ADVANCED SCIENCE
Open Access

Supporting Information

for *Adv. Sci.*, DOI 10.1002/advs.202303411

Preferential Regulation of Γ-Secretase-Mediated Cleavage of APP by Ganglioside GM1 Reveals a Potential Therapeutic Target for Alzheimer's Disease

Xiaotong Wang, Rui Zhou, Xiaqin Sun, Jun Li, Jinxin Wang, Weihua Yue, Lifang Wang, Hesheng Liu, Yigong Shi and Dai Zhang**

Supporting information

Preferential Regulation of γ-secretase-mediated Cleavage of APP by Ganglioside GM1 Reveals a Potential Therapeutic Target for Alzheimer's Disease

Xiaotong Wang[1,2#], Rui Zhou[3#], Xiaqin Sun[1#], Jun Li[1], Jinxin Wang[4], Weihua Yue[1, 5], Lifang Wang[1], Hesheng Liu[2,6], Yigong Shi[3,7,8,9], Dai Zhang[1,2*]*

[1]Peking University Sixth Hospital, Peking University Institute of Mental Health, NHC Key Laboratory of Mental Health (Peking University), National Clinical Research Center for Mental Disorders (Peking University Sixth Hospital), Beijing, China.

[2]Changping Laboratory, Beijing, China.

[3]Beijing Frontier Research Center for Biological Structure, Tsinghua-Peking Joint Center for Life Sciences, School of Life Sciences, Tsinghua University, Beijing 100084, China.

[4]State Key Laboratory of Cognitive Neuroscience and Learning and IDG/McGovern Institute for Brain Research, Beijing Normal University, Beijing, China.

[5]PKU-IDG/McGovern Institute for Brain Research, Peking University, Beijing, China.

[6]Biomedical Pioneering Innovation Center, Peking University, Beijing, China.

[7]Westlake Laboratory of Life Science and Biomedicine.

[8]Key Laboratory of Structural Biology of Zhejiang Province, School of Life Sciences, Westlake University.

[9]Institute of Biology, Westlake Institute for Advanced Study, 18 Shilongshan Road,

Xihu District, Hangzhou 310024, Zhejiang Province, China.

#These authors contributed equally to this study.

*To whom correspondence should be addressed.

Rui Zhou

zr2020@tsinghua.edu.cn

Dai Zhang

daizhang@bjmu.edu.cn

This PDF file includes:

Supporting Figures 1 to 4

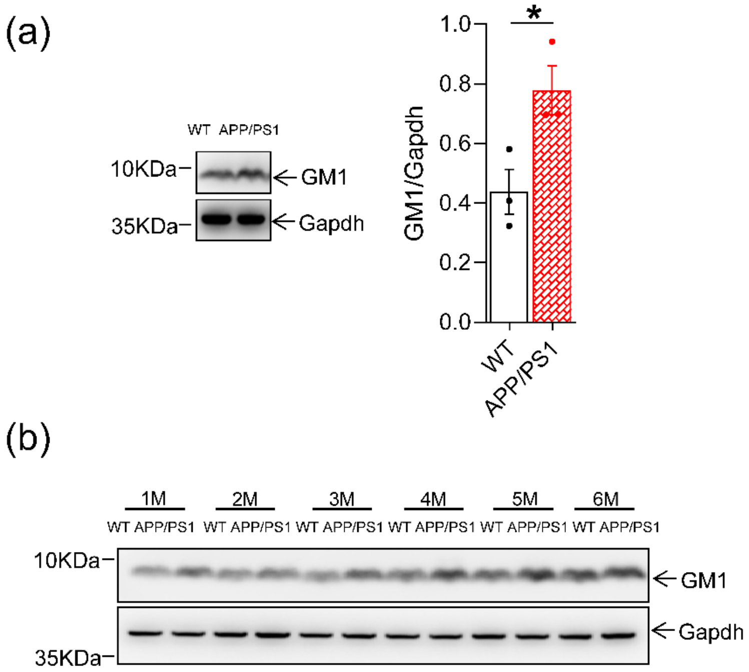

Figure S1. GM1 levels in the hippocampus of the APP/PS1 mice were higher, and further increased with age. (a) Western blotting analysis of GM1 levels in 8-month-old mice (n = 3 per group). The data were presented as means ± SEM. Two-tailed Student's t-test was performed. *p<0.05 versus WT. (b) Western blotting analysis of GM1 levels in mice aged from 1 to 6 months. APPswe/PS1ΔE9 (APP/PS1).

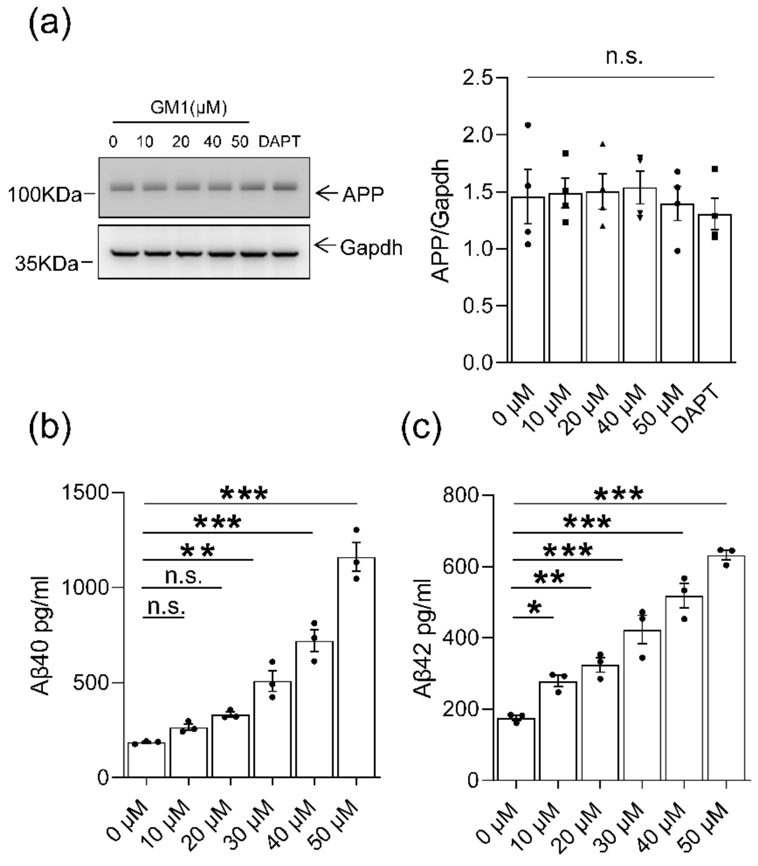

Figure S2. GM1 specifically upregulates Aβ generation but does not alter the APP levels. (a) Western blotting analysis of APP levels in N2aAPP695 cells. N2aAPP695 cells were treated with the indicated concentrations of GM1 for 8 h followed by endogenous APP detection by using western blot, APP levels were normalized to Gapdh (n = 4 per group). (b-c) ELISA examination of Aβ40/42 levels in the medium of N2aAPP695 cells treated with vehicle or different concentration of GM1 for 8 h (n = 3 per group). The data were presented as means ± SEM. one-way ANOVA with Tukey's post hoc test was performed. *p < 0.05, **p < 0.01, ***p < 0.005. ns, not significant. β-amyloid peptides (Aβ), amyloid β-protein precursor (APP), Enzyme-linked immunosorbent assay (ELISA).

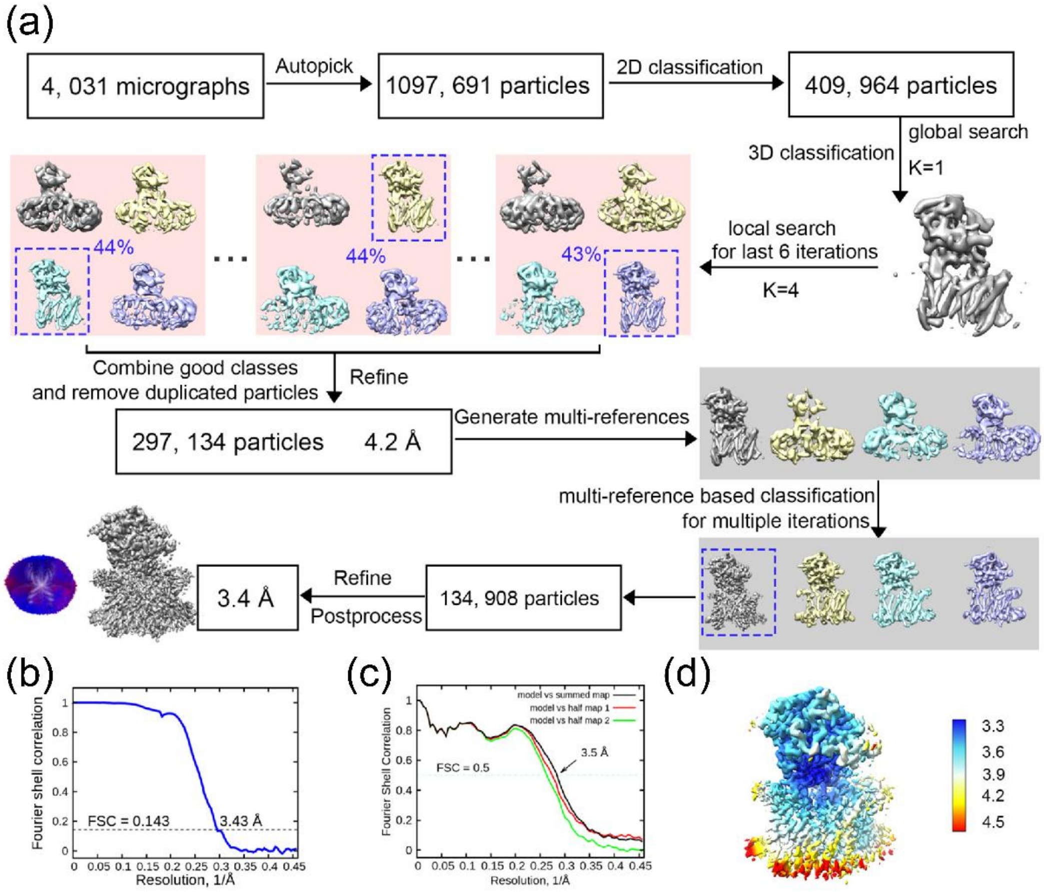

Figure S3. Cryo-EM analysis of human γ-secretase bound to GM1.(a) The flowchart for cryo-EM data processing. (b) The final average resolution of γ-secretase in the presence of GM1 is estimated to be 3.4 Å based on the 0.143 FSC curve. (c) The FSC curves of the refined model versus the maps that it is refined against (black); the model refined in the first of the two independent maps used for the FSC calculation versus that same map (red); and the model refined in the first of the two independent maps versus the second independent map (green). The small difference between the red and green curves indicates that the refinement did not suffer from overfitting. (d) Color-coded local resolution distribution in Å of the final reconstruction, estimated by RELION-3.0. Cryo-electron microscopy (Cryo-EM), Fourier shell correlation (FSC).

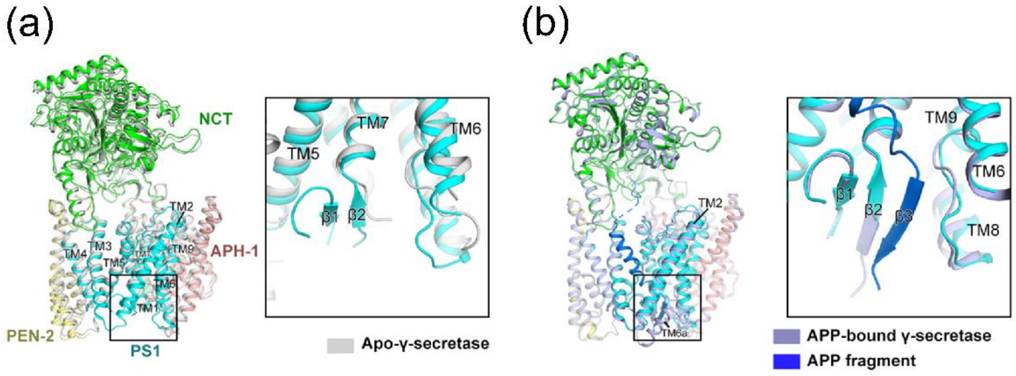

Figure S4. Structural comparison among γ-secretase bound to GM1 (four subunits differentially colored), apo-γ-secretase (grey) and γ-secretase in complex with APP fragment (light purple and marine). (a) Structural alignment of γ-secretase bound to GM1 and apo-γ-secretase (grey, PDB ID: 5a63). TM2 of PS1 becomes ordered upon the addition of GM1 (left panel). Meanwhile, the β-sheet (β1 and β2) forms at the intracellular side after GM1 treatment. (b) γ-secretase with GM1 shares similar features to γ-secretase in complex with APP fragment (PDB ID: 6iyc). Superimposition of two γ-secretase molecules leads to an r.m.s.d. (root mean square deviation) of 0.375 Å for aligned 1129 Cα atoms. β1 and β2 adopt a nearly identical conformation in two γ-secretase molecules. Based on γ-secretase-APP complex structure, another β-strand from substrate (β3) will form alongside β2 before cleavage.TM6a in γ-secretase bound to GM1 was not built due to the lack of continuous local density. Transmembrane (TM).
